# The role of robotic surgery for the treatment of hilar cholangiocarcinoma: A systematic review

**DOI:** 10.3389/fonc.2022.1001838

**Published:** 2022-09-28

**Authors:** Alberto Brolese, Marta Rigoni, Alessio Pasquale, Giovanni Viel, Marco Brolese, Francesco Antonio Ciarleglio

**Affiliations:** ^1^ Department of General Surgery and Hepato-Pancreato-Biliary (HPB) Unit – APSS, Trento, Italy; ^2^ Department of Biomedical, Surgical and Dental Sciences, Faculty of Medicine and Surgery, University of Milan, Milan, Italy; ^3^ Department of Surgery, University of Padua, Padua, Italy

**Keywords:** hilar cholangiocarcinoma, Klatskin tumor, robotic liver resection, liver resection, biliary tumor

## Abstract

**Background:**

The role of robotic surgery (RS) for hilar cholangiocarcinoma (HC) is under investigation. Surgical resection is the only curative modality of treatment but extremely complex and high risk of morbidity and mortality may occur. The aim of this study is to perform a systematic review of perioperative and oncological outcomes of RS for HC, across a comprehensive range of outcomes reported in recent literature.

**Materials and Methods:**

PRISMA checklist was used as a basis for writing the systematic review and studies’ selection. Literature documenting RS for HC was analyzed by searching PubMed and Cochrane Library from 2009 to May 2022. The search terms, either independently or in combination, were used according to PICOT framework. The target population are patients treated with robotic surgical approach for HC.

**Results:**

12 studies with 109 patients were included after screening process. The Bismuth classification in all series except one was: 21 type I, 7 type II, 12 type IIIa, 26 type IIIb and 4 type IV. Mean operative time for a total of 21 patients was 644 minutes. Other two case series reported a median operative time of 375 with a console time of 276 minutes. Mean blood loss for case reports and two case series was 662 milliliters. Blood transfusion rate for all operation was 33.3%. Overall Conversion rate was 2.8%. Pooled post operative morbidity and mortality was 39.8% and 1.8% respectively. Mean LOS for case reports and one case series for a total of 17 patients was 16 days. R0 resection rate for the 11 papers was 74.3%. Seven out of 12 studies reported on the oncological follow up: median observation time ranged from 5 to 60 months, recurrence rate was 52.6% (range 0-90%) reported only in 19 patients (10/19).

**Conclusions:**

RS for HC was feasible and safe. However, although this systematic review could not be conclusive in most of the analyzed items, RS for the treatment of HC could represent the best tool for a future meticulous and precision surgery. The review’s results certainly indicate that further research in urgently is required on this field.

## Introduction

Biliary malignancies are the second most prevalent primary liver neoplasia, following hepatocellular carcinoma, and their incidence is on the rise, with an estimated 1.8% annual increase (1). Hilar cholangiocarcinoma (HC) is a rare hepatobiliary malignancy, with an incidence of 1.2 cases/100,000 people in Western countries (2). HC, also referred to as Klatskin tumor (KT), arises from the epithelial cells of the bile ducts and presents a highly aggressive behavior with a high propensity for vascular, perineural, and liver invasion. Due to the late onset of symptoms and lack of effective non-surgical treatments, the mean disease-specific survival is still less than 1 year after diagnosis (2).

Surgical resection with curative intent has been recognized as the primary therapy and sole procedure for curing patients with resectable local disease (3). Oncological biliary tract resection is one of the most challenging abdominal procedures, with high rates of major morbidity and recurrence. Less bleeding, minimal liver damage, and a good oncological outcome are the cornerstones for the treatment of HC. The optimal surgical treatment for an oncological resection of HC is radical extrahepatic bile duct resection in conjunction with major hepatectomy, radical lymphadenectomy, and Roux-en-Y hepaticojejunostomy reconstruction (4, 5).

Robotic surgery (RS) has recently emerged as an alternative for minimally invasive liver surgery; however, its role in biliary tract cancer remains unclear. RS will find its place in hepatobiliary surgery since it can facilitate the most technically challenging procedures such as biliary anastomoses. A robotic approach has been introduced to overcome some of the typical limitations of laparoscopy, including a wider range of movements and enhanced instrument dexterity, a three-dimensional view of the surgical field, a reduction in surgeon tremors, and a shorter learning curve. RS has demonstrated promising results in terms of morbidity, mortality, length of hospital stay, and postoperative recovery in a subset of patients (6). Furthermore, an improvement in perioperative outcomes could have an impact on oncological results, thereby improving long-term survival and recurrence rates. The role of RS in HC remains a subject of discussion, as it is still debatable whether it can produce optimal and appropriate results. In the past 12 years, only a few case reports or small single-center case series have examined the efficacy of RS in the surgical treatment of HC.

The aim of this study is to conduct a comprehensive systematic review of the perioperative and oncological outcomes of RS for HC, as reported in recent literature. The ultimate objective is to demonstrate RS’ state of the art, while taking into account safety, feasibility, and efficacy in this new frontier of treatment for KT.

## Materials and methods

### Literature search strategy

The Preferred Reporting Items for Systematic Reviews and Meta-Analyses (PRISMA) checklist formed the basis for writing the systematic review, and the PRISMA flow diagram was used to report the selection of studies (7). All steps were performed independently by two authors (FAC and MR). Final decisions on eligibility were resolved by consensus.

**Figure 1:**
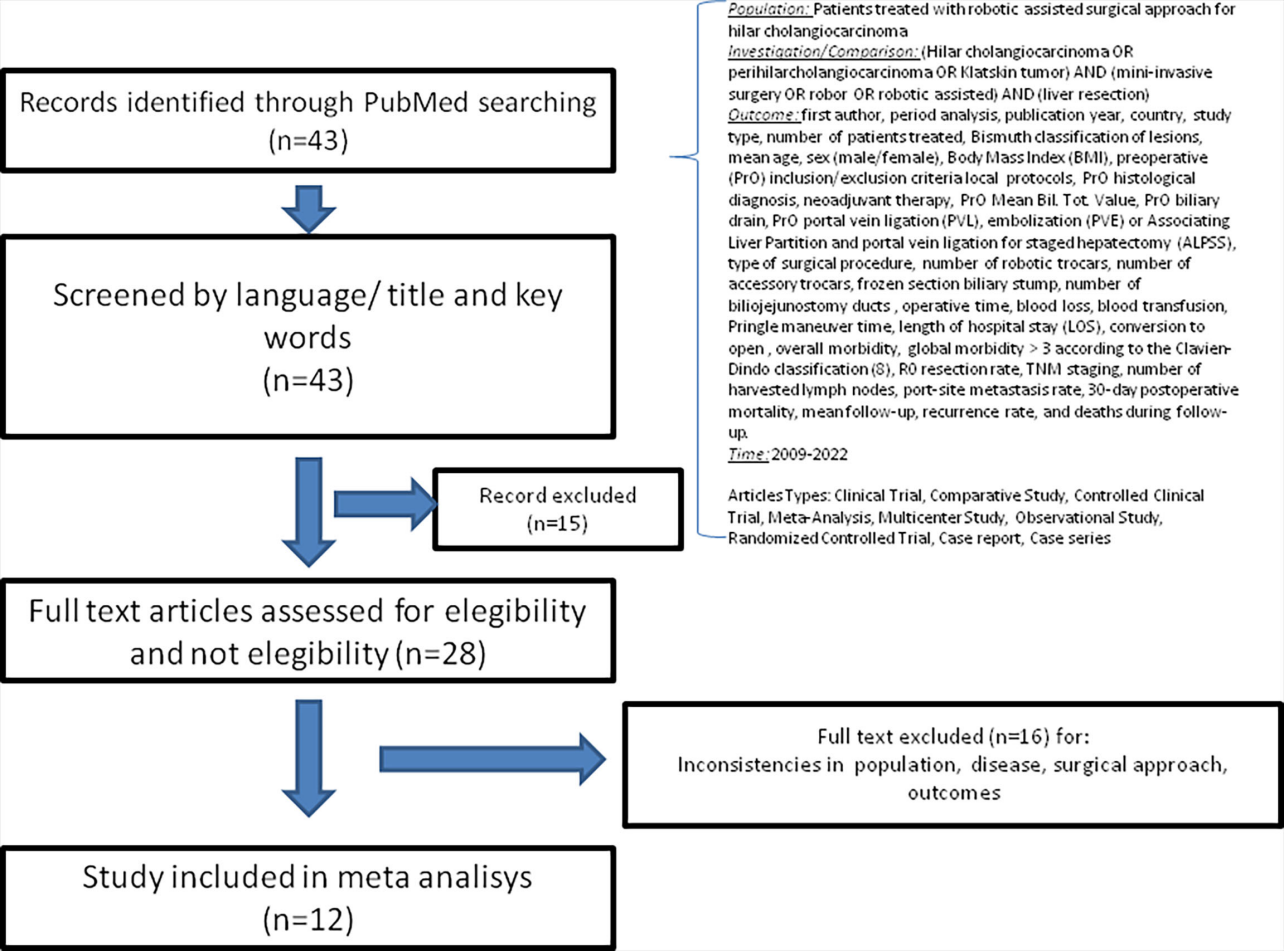
Flow diagram depicting the systematic review’s article selection process based on the PRISMA diagram (7).

The literature documenting RS for HC was analyzed by searching PubMed and The Cochrane Library from 2009 to May 2022. Independent or combination search terms were used according to the PICOT framework (**Figure 1**). The keywords or combinations used were as follows: (hilar cholangiocarcinoma OR perihilar cholangiocarcinoma OR Klatskin tumor) AND (minimally invasive surgery OR robot OR robotic-assisted surgery) AND (liver resection). A methodical search was conducted for relevant systematic reviews, randomized controlled trials, observational studies (prospective or retrospective cohort and case–control or matched case–control studies), case series, and reports using a search strategy guided by oncological or surgical information, abstract, and keywords related to our research question. Only published articles in the English language were screened. With the exception of multicenter studies, articles with the largest series or the most recent publication date were selected when more than one article was reported by the same institution or author.

### Study inclusion criteria

The titles and abstracts of all the studies were screened and a full text assessment was then conducted. Patients treated for HC with robotic liver resection surgery with or without biliary tract resection, cholecystectomy, and lymphadenectomy were the target population. Studies were eligible if they included an evaluation and report on one of the perioperative or oncological outcomes of the robotic resection performed for HC. All types of resections for KT were included. The PICOT (Population, Intervention, Comparison, Outcome, and Time) framework (**Figure 1**) was used to define the study selection criteria. The following studies or data were excluded: robotic surgical procedures for staging or palliative care, those that lacked HC cases in the results, and the lack of a robotic approach, abstracts, editorials, or reviews. The quality of the primary studies was not a criterion for exclusion.

### Outcomes

The following data, clinical, and oncological outcomes were collected: first author, period analysis, publication year, country, study type, number of patients treated, Bismuth classification of lesions, mean age, sex (male/female), body mass index (BMI), preoperative (PrO) inclusion/exclusion criteria local protocols, PrO histological diagnosis, neoadjuvant therapy, PrO Mean Bil. Tot. Value, PrO biliary drain, PrO portal vein ligation (PVL), embolization (PVE) or Associating Liver Partition and portal vein ligation for staged hepatectomy (ALPSS), type of surgical procedure, number of robotic trocars, number of accessory trocars, frozen section biliary stump, number of biliojejunostomy ducts, operative time, blood loss, blood transfusion, Pringle maneuver time, length of hospital stay (LOS), conversion to open, overall morbidity, global morbidity > 3 according to the Clavien–Dindo classification (8), R0 resection rate, TNM staging, number of harvested lymph nodes, port-site metastasis rate, 30-day postoperative mortality, mean follow-up, recurrence rate, and deaths during follow-up.

### Data extraction and quality assessment

Two reviewers (FAC and MR) independently screened the titles, abstracts, and full texts of the selected studies and extracted demographic and clinical outcome data. In the case of disagreement, they reviewed the papers together to reach consensus. The methodological quality of the studies was evaluated using the Oxford Centre for Evidence Medicine’s critical appraisal tool, checklists of the Dutch Cochrane Centre, BMJ editor’s checklists, and the checklists of the EPPI Centre (9). The overall quality of the primary studies was rated as either very low, low, moderate, or high.

### Statistical analyses

All of the analyses were conducted using data from the included studies. When available, patient characteristics and outcomes were reported as numbers or percentages, mean ± standard deviation, or median (interquartile range or range), as reported in the primary studies.

To provide a pooled estimate of the outcomes, we calculated the total percentages of dichotomous outcomes by adding the numbers of events and patients from the original primary studies. Some of the included studies reported continuous outcomes with means and no standard deviations, while others reported medians and ranges. These heterogeneities in the effect measure prevented us from combining certain outcomes globally (operative time, blood loss in milliliters, Pringle time in minutes, number of lymph nodes, and length of stay). However, for such outcomes, we calculated the mean of case reports and case series that reported outcomes for individual patients. Moreover, due to the lack of data across studies, we were unable to provide a pooled estimate of the port site. Analyses were carried out using Stata and Excel software.

## Results

### Study characteristics and population

The flowchart in **Figure 1**, which depicts the selection of articles for the systematic review, is based on the PRISMA diagram. The initial search returned 43 English-language results. After examining the titles and keywords, 15 citations were eliminated as they were deemed irrelevant. Twenty-eight studies were assessed for eligibility through full-text evaluation. Due to inconsistencies in population, disease, surgical approach, or outcomes, 14 records were removed following full-text screening. The remaining 12 studies (10–21) were included in the quantitative synthesis of this systematic review. Eight case reports (10, 12, 14, 16, 17, and 19-21) and four case series (11, 13, 15, and 18) describing only single-center RS experiences for HC were selected. Four were from China, three from Italy, three from the US, one from Brazil, and one from Spain. No randomized controlled trials were retrieved. All the results are summarized in **Tables 1** and **2**. **Table 3** details the quality assessment of each included study. All studies were deemed to be of low or very low quality.

**Table 1:** Studies on robotic surgery for HC: study, patients, and procedural characteristics.

Study	Year	Study period	Study type	No. of patients	Bismuth type	Age (years)	Male/Female	BMI	Inclusion/exclusion criteria	Pre op biopsy positive	Neadj therapy	Bil Tot value	Pre op biliary drain (*n*, %)	Pre op PVE/PVL/ALPSS (*n*, %)
Giulianotti et al. (10)	2010	2009	Case Report	1	3a	66	1M	23	na	1/1 (100%)	no	na	1/1 (100%)	1 PVE
Liu et al. (11)	2012	2009–2011	Case series	39	na	na	na	na	na	na	na	na	na	na
Zhu et al. (12)	2014	2011	Case Report	1	IIIa	43	M	na	na	na	na	23 mmol	1/1 (100%)	PVE
Xu et al. (13)	2016	2009–2012	Case series	10	1 type II/1 type IIIb/4 type IIIa/4 type IV	57.6*;54**	8M/2F	na	na	na	na	145.6 mmol/L *	6/10 (60%)	na
Quijano et al. (14)	2016	2011–2014	Case Report	1	IIIb	na	na	na	na	na	na	na	na	na
Li et al. (15)	2020	2017–2019	Case series	48	20 type I/6 type II/5 type IIIa/17 type IIIb	62.9*	28M/20F	2.7*	na	na	0/48 (0%)	30 pts (62.5%)	na	na
Machado et al. (16)	2020	2019	Case Report	1	IIIb	76	F	30	na	1/1 (100%)	1/1 (100%)	na	1/1 (100%)	na
Marino et al. (17)	2020	2019	Case Report	1	IIIb	57	1M	na	na	na	na	5.2 mg/dl	na	na
Cillo et al. (18)	2021	2019–2020	Case series	4	4-3b	60.5*	1M/3F	na	Y	3/4 (75%)	1/4 (25%)	4.67 *	4/4 (100%)	0/4 (0%)
Sucandy et al. (19)	2021	2020	Case Report	1	IIIb	77	1F	na	na	1/1 (100%)	na	na	1/1 (100%)	na
Di Benedetto et al. (20)	2022	2021	Case Report	1	3a	74	1F	na	na	1/1 (100%)	no	na	na	1 ALPSS + PVE
Tee et al. (21)	2022	2021	Case Report	1	I	58	M	49		1/1 (100%)	0/1 (0%)	na	1/1 (100%)	na

ALPSS, associating liver partition and portal vein ligation for staged hepatectomy; BMI , body mass index; F , female; HC , hilar cholangiocarcinoma; M , male; na, not available; PVE , portal vein embolization; PVL, portal vein ligation; * , mean; ** , median. na, not applicable.

**Table 2:** Studies on robotic surgery for HC: study, patients, and procedural characteristics.

Study	Surgery	Nr Rob trocars	Nr Acc Trocar	Frozen section bil stump	Nr ducts of biliary anasto	Operative time (min)	Blood loss (ml)	Blood trasfusion	Pringle time (min)	LOS (days)	Conversion to open (*n*, %)	Global morbidity (*n*, %)	Morbidity >3 Clavien–Dindo	R0 res rate (*n*, %)	TNM	Number of lymph nodes	Port site metastasis (*n*, %)	Post-operative death (*n*, %)	Follow-up (months)	Recurrence rate (*n*, %)	Deaths during follow-up
Giulianotti et al. (10)	Right ext Hep+S1+limphadenectomy+biliary carrefour res+hepatico-jejunostomy	4	1	1/1 (100%)	1	540	800	1/1 (100%) with 1 EC	0/1 (0%)	11	0/1 (0%)	0/1 (0%)	0/1 (0%)	1/1 (100%)	T2N0	na	na	0/1 (0%)	8	0/1 (0%)	0/1 (0%)
Liu et al. (11)	L Hep 3/biliary res and hepjejunostomy 15/others 21	4	2	na	na	355*; 375**	na	na	na	na	1/39 (2.6%)	1/39 (2.6%)	na	na	na	na	1/39 (2.6%)	1/39 (2.6%)	na	na	na
Zhu et al. (12)	Right Hep+S1+lymphadenectomy+biliary carrefour res+hepatico-jejunostomy	na	0/1 (0%)	1/1 (100%)	na	na	700	na	na	14	0/1 (0%)	0/1 (0%)	0/1 (0%)	1/1 (100%)	na	na	na	0/1 (0%)	na	na	na
Xu et al. (13)	R (5)/L (4)/trisegm Right(1)Hep enl Sg1, biliary tract res and lymphadenectomy	4	2	8/10 (80%)	na	703*	1360*	6/10 (60%)	na	16 **	0/10 (0%)	9/10 (90%)	3/10 (30%)	7/10(70%)	na	na	na	1/10 (10%)	60	9/10 (90%)	na
Quijano et al. (14)	Left Hep+S1+lymphadenectomy+biliary carrefour res+hepatico-jejunostomy	4	2	1/1 (100%)	na	510	1000	na	na	16	1/1 (100%)	1/1 (100%)	0/1 (0%)	1/1 (100%)	T2N0	13	na	0/1 (0%)	na	na	na
Li et al. (15)	R/L Hep enl Sg1, biliary tract res and lymphadenectomy	4	1	48/48 (100%)	na	276** (only console time)	150*	13/48 (27.1%)	14/48 (29,2%)	9**	0/48 (0%)	28/48 (58.3%)	5/48 (10.4%)	35/48 (72.9%)	na	na	na	0/48 (0%)	na	na	na
Machado et al. (16)	Left Hep+S1+lymphadenectomy+biliary carrefour res+hepatico-jejunostomy	4	1	1/1 (100%)	1	480	740	2 ec	na	na	0/1 (0%)	1/1 (100%)	0/1 (0%)	1/1 (100%)	T1aN0	na	na	0/1 (0%)	5	0/1 (0%)	0/1 (0%)
Marino et al. (17)	Left Hep+S1+lymphadenectomy+biliary carrefour res+hepatico-jejunostomy	4	2	1/1 (100%)	1/1 (100%)	295	280	na	0/1 (0%)	6	0/1 (0%)	0/1 (0%)	0/1 (0%)	1/1 (100%)	na	9	na	0/1 (0%)	na	Na	na
Cillo et al. (18)	Left Hep+S1+lymphadenectomy+biliary carrefour res+hepatico-jejunostomy	4	4	4/4 (100%)	2,75*	850*	840*	0/4 (0%)	19,25*	9**	1/4 (25%)	3/4 (75%) bil first grade A, ileous, asympt segm ileous ischemia	0/4 (0%)	3/4 (75%)	T3Nx/T2aN1/T4N1/T4N0	>14 on 3/4 and 0 on 1 pt	na	0/4	7,5**	1/4 (25%)	0/4 (0%)
Sucandy et al. (19)	Left Hep+S1+lymphadenectomy+biliary carrefour res+hepatico-jejunostomy	na	na	1/1 (100%)	1	360	na	na	na	6	0/1 (0%)	na	na	1/1 (100%)	na	na	na	0/1 (0%)	12	0/1 (0%)	0/1 (0%)
Di Benedetto et al. (20)	Right ext Hep+S1+lymphadenectomy+biliary carrefour res+hepatico-jejunostomy	4	2	1/1 (100%)	2	370 (previous ALPSS)	450	0/1 (0%)	0/1 (0%)	19	0/1 (0%)	0/1 (0%)	0/1 (0%)	0/1 (0%)	T4N1	21	na	0/1 (0%)	13	0/1 (0%)	0/1 (0%)
Tee et al. (21)	bile duct res, cholecystectomy, hilar lymph and roux hep jejunostomy	5	3	1/1 (100%)	1	540	100	0/1 (0%)	na	5	0/1 (0%)	0/1 (0%)	0/1 (0%)	1/1 (100%)	T2N1M0	12	na	0/1 (0%)	12	0/1 (0%)	0/1 (0%)

ALPSS, associating liver partition and portal vein ligation for staged hepatectomy; LOS, length of stay; *, mean; **, median. na, not available.

**Table 3:** Critical appraisal of the included studies based on the Critical Appraisal tool of the Center for Evidence-Based Management-CENMa (9).

Study	Year	Did the study address a clearly focused question/issue?	Is the research method (study design) appropriate for answering the research question?	Are both the setting and the subjects representative with regard to the population to which the findings will be referred?	Is the researcher’s perspective clearly described and taken into account?	Are the methods for collecting data clearly described?	Are the methods for analyzing the data likely to be valid and reliable? Are quality control measures used?	Was the analysis repeated by more than one researcher to ensure reliability?	Are the results credible, and if so, are they relevant for practice?	Are the conclusions drawn justified by the results?	Are the findings of the study transferable to other settings?	Overall level, and quality of evidence
Giulianotti et al. (10)	2010	Yes	Cannot tell	Yes	Yes	Yes	No	Yes	Yes	Yes	Yes	Level IV, very low quality
Liu et al. (11)	2012	Yes	No	Cannot tell	Yes	No	No	Cannot tell	Yes	Yes	Yes	Level IV, very low quality
Zhu et al. (12)	2014	Yes	Cannot tell	Yes	Yes	Yes	No	Yes	Yes	Yes	Yes	Level IV, very low quality
Xu et al. (13)	2016	Yes	Yes	Yes	Yes	Yes	No	Yes	Yes	Yes	Yes	Level IV, low quality
Quijano et al. (14)	2016	Yes	Yes	Cannot tell	Yes	Yes	No	Yes	Yes	Yes	Yes	Level IV, very low quality
Li et al. (15)	2020	Yes	Yes	Yes	Yes	Yes	Yes	Yes	Yes	Yes	Yes	Level IV, low quality
Machado et al. (16)	2020	Yes	Yes	Yes	Yes	Yes	No	Yes	Yes	Yes	Yes	Level IV, low quality
Marino et al. (17)	2020	Yes	Yes	Yes	Yes	Yes	No	Yes	Yes	Yes	Yes	Level IV, very low quality
Cillo et al. (18)	2021	Yes	Yes	Yes	Yes	Yes	Yes	Yes	Yes	Yes	Yes	Level IV, low quality
Sucandy et al. (19)	2021	Yes	Yes	Yes	Yes	Yes	No	Yes	Yes	Yes	Yes	Level IV, very low quality
Di Benedetto et al. (20)	2022	Yes	Yes	Yes	Yes	Yes	No	Yes	Yes	Yes	Yes	Level IV, low quality
Tee et al. (21)	2022	Yes	Yes	Yes	Yes	Yes	Yes	Yes	Yes	Yes	Yes	Level IV, low quality

The analysis included a total of 109 patients, with the largest study reporting a series of 48 cases (15). Except for one case report (12), all the subjects in this review were over 54 years old. Only one study reported preoperative exclusion criteria for robotic surgical resection, along with preoperative neoadjuvant therapy, major underlying disease, Bismuth type IV, and stage beyond T4 (18). Information regarding preoperative diagnosis and preoperative biliary drainage was present in six (10, 16, and 18-21) and seven studies (10, 12, 13, 16, 18, 19, and 21), respectively. With one exception, extension of local disease was expressed according to the Bismuth classification in all series and reports (11). Except for one series (12), the Bismuth classification was as follows: 21 type I, 7 type II, 12 type IIIa, 26 type IIIb, and 4 type IV. Only four studies (10, 12, 18, and 20) were linked to PrO future liver remnant (FLR) evaluation and applied portal vein embolization or ligation or ALPSS to prevent postoperative liver failure. Robotic major liver resection enlarged to segment 1, biliary carrefour resection, and lymphadenectomy were performed in 72/109 cases (66%). With one exception (11), all studies disclosed information regarding the frozen section of the biliary stump. Only two authors (18, 20) reported more than one bile duct anastomosis.

Operative time was provided by all series except one (12). Case reports (10, 14, 16, 17, and 19-21) and two case series (13, 18) for a total of 21 patients had a mean operative time of 644 min. Another two case series reported median operative times of 375 (11) min and a console time of 276 min (15). Data on blood loss and blood transfusion rate were reported in all series except two (11, 19). The mean blood loss for case reports (10, 12, 14, 16, 17, 20, and 21) and two case series (13, 18) for a total of 21 patients was 662 milliliters. The case series by Li et al. reported a median of 150 ml for 48 patients (15). The blood transfusion rate for all operations was 33.3% (22/66). All studies reported the conversion rate, and its global ratio was 3/109, resulting in a total percentage of 2.8%. Only one article discussed the reasons for conversion (18).

One paper failed to provide complete data on postoperative morbidity (19). The pooled postoperative morbidity rate was 43/108 (39.8%). The stratified incidence of combined morbidity for severe complications (Clavien–Dindo classification grade ≥ 3) was 8/69 (11.6%). All studies reported the postoperative mortality rate, with a pooled total of 2/109 (1.8%). Postoperative deaths were caused by abdominal infection (11) and liver failure (13).

Two studies (11, 16) did not provide data on LOS. The mean LOS for case reports (10, 12, 14, 17, and 19-21) and one case series (13) for a total of 17 patients was 16 days; the case series by Li et al. (15) reported a median of 9 days (range, 4–52) and the case series by Cillo et al. (18) also reported a median of 9 days.

Pathological TNM staging was reported in all but six studies. According to data from four studies (14, 17, 20, and 21), the mean number of harvested lymph nodes was 14. Only one study (11) failed to report data on the status of margins at the final pathological examination. The rate of R0 resection for the 11 papers analyzed was 74.3% (52/70). Seven out of 12 studies reported on the oncological follow-up: the median observation time ranged between 5 and 60 months, and the recurrence rate was 10/19 (52.6%; range, 0–90%) (10, 13, 16, and 18-21). Inadequate data and the heterogeneity of the study population or metrics employed prevented a cumulative analysis of disease and overall survival. It should be highlighted that the patients included in these case reports and series may have been clinically selected. This may limit the generalizability of results for a larger population of patients with the same disease.

## Discussion

HC is a malignant disease with a poor prognosis and survival rate (22). Even among patients with localized disease, few tumors are amenable to anatomical radical resection due to a high incidence of local invasion and proximity to hilar hepatic vessels. Thus, very aggressive surgical treatment is generally required to increase the rate of curative resection and long-term survival (23). The results of this systematic review are based on the analysis of a small number of studies with a high probability of bias. In addition, the total number of enrolled subjects is very small, and the overall experience is much lower than that of the published counterpart treated with conventional open surgery.

Both open and minimally invasive surgical approaches for HC are acknowledged to be technically challenging due to the anatomical characteristics of the hepatic hilum and the biological characteristics of cholangiocarcinoma, which requires clean radial margins for curative resection. The complexity of these procedures stems from the need for precise hilar dissection and lymphadenectomy, as well as biliary reconstruction, which has primarily acted as a barrier to the propagation of the laparoscopic technique. Compared to minimally invasive surgery for hepatocellular carcinoma, minimally invasive resection for HC is a relatively new field in HPB surgery. However, the rapid development of surgical expertise and equipment has increased the use of laparoscopic and robotic techniques to treat all Bismuth classification grades of KT (24). In 2010, Giulianotti et al. (10) used the Da Vinci Robotic Surgical System (Intuitive Surgical, Sunnyvale, CA, USA) to perform an extended right hepatectomy, biliary carrefour resection, and left hepaticojejunostomy for HC. Their experience demonstrated the technical feasibility of a robotic approach to HC. Among the advantages cited by the authors, the robotic platform provides surgeons with three-dimensional stereoscopic visualization, and EndoWrist enables surgeons to perform various complicated and challenging maneuvers, including biliary anastomoses, with greater stability than traditional laparoscopic instruments (25). In recent years, interest in and reliance on robotic approaches for treating lesions classified as Bismuth grades I–III have increased as a result of these initial experiences. In this review, we reported 71 major enlarged caudate lobe liver resections on 109 patients (65.1%). Robotic surgery is best suited for procedures requiring high level precision and dexterity, and the reconstructive phase is where the majority of technical benefits are reported. Our data demonstrated that robotic-assisted treatment of HC is feasible and reproducible. In particular, the curative intent of KT treatment has been maintained. However, only one of the case series included in this review specified inclusion and exclusion criteria as well as restrictive allocation criteria for the robotic approach (18). These results are indicative of a clear selection bias among the patients enrolled in all the studies under consideration. Xu et al. reported robotic liver resection for enlarged caudate lobe, lymphadenectomy, resection of the extrahepatic bile duct, and Roux-en-Y hepaticojejunostomy to treat Bismuth type-IV HC in four patients (13). It was concluded that resection indications for Bismuth type-IV HC should be determined with caution because trisectionectomy was associated with more uncontrollable accidents due to the extreme complexity of the procedures (13). The paper did not provide a way to split the outcome of KT type IV from the other types. In the context of the curative intention-to-treat program, it could not be ruled out that many KT type IV patients received suboptimal oncological surgical treatment due to restrictive allocation criteria for minimally invasive procedures. Due to the biological nature of cancer, restrictive selection criteria are necessary irrespective of the surgical approach (robotic or laparoscopic). Complex Roux-en-Y hepaticojejunostomy with single or multiple bile ducts were rarely described in a minimally invasive setting. The robotic approach, with its degree of freedom and stability, could be the best way to circumvent all laparoscopic limitations. One author performed biliary reconstruction of multiple bile stumps with a robotic technique in 75% of cases, and their paper confirmed that the robotic approach was the absolute novel opportunity to also perform minimally invasive hepaticojejunostomies even when multiple ducts are present (18).

No intraoperative accidents were reported, and the overall conversion rate in this review was 2.8%. These data are more favorable than the 10% reported by the best high-volume center series regarding minimally invasive liver resection (26). The shorter learning curve for robotic-assisted surgery compared to conventional laparoscopic surgery may likely account for these results (27). A recent meta-analysis focusing on distal pancreatic resections also confirmed this effect (28).

In this review, the mean operative time and mean blood loss were highly variable. The duration of robotic surgery is typically longer than that of an open or laparoscopic approach. However, the longer operative time is still under investigation. These findings are probably due to the fact that the learning curve for HC is still in its infancy. Chen et al. observed improvements in operative time after 52 cases of robotic major hepatectomy (29). Li et al. reported 48 cases of radical robotic resection for HC, with a median operative time that only takes into account the console time (276 min; range, 170–500 min) and a mean blood loss of 150 ml (range, 20–1,500 ml) (15). In 2020, Ratti et al. compared 16 cases of laparoscopic resection versus 32 cases of open approach for HC (30). In that study, the operative time, blood loss, and transfusion rate in the laparoscopic group vs. the open group were 360 ± 290 min vs. 275 ± 200 ml and 380 ± 250 ml vs. 470 ± 390 ml, respectively. In this review, the total blood transfusion rate for all operations was 33.3%. In comparative studies by Zhang et al., the estimated blood loss and incidence rate of blood transfusion were 620.0 ± 681.2 ml and 57%, respectively (31). These data suggest that the robotic approach could further facilitate a precise and effective control of intraoperative bleeding.

This review reported good results in terms of morbidity and mortality. Pooled postoperative global morbidity was 39.8%, whereas morbidity stratified by severity according to the Clavien–Dindo classification ≥3 was 11.6%. The average pooled morbidity and mortality rates reported by open series were 14%–75% and 0%–17%, respectively (32). These data may indicate that the HC robotic approach is the final challenge in the learning curve, leading to improved outcomes.

Better survival rates depend on oncological outcomes. The overall analysis reveals a pooled R0 rate of 74.3%, which may be a satisfactory outcome when compared to large series of open surgery for HC (33). There was a paucity of data on survival and recurrence, and these data were unfortunately based on short follow-up and observation periods. Currently, information on postoperative HC recurrence is limited. Seven out of 12 studies reported on the oncological follow-up leading to a total recurrence percentage of 52.6%, ranging from 0%–90% (10, 13, 16, and 18-21). Lu et al. reported a 66.5% tumor recurrence rate after open resection with a median follow-up of 22.7 months (34).

The first report concerning the robotic approach for the treatment of HC was published in 2010 (10), 8 years after the first paper on robotic liver resection (35). In their review, Cipriani et al. reported fewer than 200 minimally invasive procedures (laparoscopic and robotic) for HC (32). The latter approach and its slow propagation are coincident with the technical challenges involved and the low resectable rate of KT. None of the centers that have dealt with RS in KT have identified any absolute contraindications to the robotic approach. The benefits of suturing in biliojejunostomy anastomosis and the advantageous possibility of performing liver resection in a restricted space have been identified with the use of the robotic approach. Different centers, different skills in HPB surgery, and substantial bias in patient selection and surgical procedures influence the final black-and-white results. Presently, the potential benefits in terms of short-term and oncological outcomes are only theoretical and must be investigated through a comparative study of laparoscopic and open approaches. This review supports the feasibility and efficacy of RS for HC after assessing surgical and oncological outcomes. The next step could be a multicentric comparative study to validate and strengthen the results. Randomized controlled trials will be necessary to further confirm this hypothesis.

### Study limitations

This systematic review has several limitations. First, the literature search was carried out by only consulting the two most relevant scientific databases for medical practice (PubMed and The Cochrane Library). Second, the review was limited by the lack of randomized controlled trials, large observational cohort studies, and comparative studies in general. In fact, the totality of studies we found were case reports and case series. As a result, the quality of the included studies was rated as low or very low, limiting the strength and reliability of our results; however, a recent study has demonstrated that it is possible to write rigorous clinical practice guidelines and recommendations for rare diseases or areas where there is little or low- or very-low-quality evidence (36). Due to the absence of a control group, we were unable to conduct a comparative meta-analysis of outcomes and could only perform a descriptive pooled estimation on a subset of outcomes. Moreover, we combined results from the case reports (i.e., involving 1 patient) with a case series that involved a larger number of patients (4, 10, 39, and 48) without weighing the data. Finally, we were unable to statistically investigate the heterogeneity of studies. Different patient demographic characteristics (13, 14) and surgical intervention characteristics (11, 14) were the clinical heterogeneity sources in this review. It must be stated that, as robotic surgery is still in its infancy, the patients included in this review (i.e., in the primary studies, case reports, and series) may have been clinically selected patients. This may limit the generalizability of results and necessitate the application of this technique to a wider audience of patients with the same disease.

### Implications for future research

Our systematic review provides preliminary evidence on oncological RS for HC. The review’s results certainly indicate that this topic urgently requires additional research. Particularly, it would be of utmost importance to increase the number of patients (and the number of studies) on this topic, as well as to generate evidence of higher methodological quality in terms of study design, execution, and the reporting of findings.

## Conclusion

Despite the fact that this systematic review was inconclusive, RS for the treatment of HC could certainly represent the best tool for future meticulous and precise surgery that is currently only possible with expert hands and extensive skill with liver RS. To treat a disease as particular as KT, it is necessary to consider a number of specific aspects, including patient and center characteristics, organizational factors, and team acceptance. The main criticism in the majority of series is the very long operative time. However, if surgery must become increasingly precise today, RS for the treatment of HC may become one of the best indications and potentially the most suitable tool for quality surgery.

## Data availability statement

The original contributions presented in the study are included in the article/supplementary material. Further inquiries can be directed to the corresponding author.

## Author contributions

AB, MR and FAC contributed equally to this work and share first authorship. AB, MR and FAC contributed to conception and design of the study. FAC organized the database. MR performed the statistical analysis. AB and FAC wrote the first draft of the manuscript. AB, MR, AP, GV, MB and FAC wrote sections of the manuscript. AP, GV, and MB contributed equally to this work about data analysis and revision. All authors contributed to manuscript revision, read, and approved the submitted version.

## Conflict of interest

The authors declare that the research was conducted in the absence of any commercial or financial relationships that could be construed as a potential conflict of interest.

## Publisher’s note

All claims expressed in this article are solely those of the authors and do not necessarily represent those of their affiliated organizations, or those of the publisher, the editors and the reviewers. Any product that may be evaluated in this article, or claim that may be made by its manufacturer, is not guaranteed or endorsed by the publisher.
